# A comparative study of orthotropic and isotropic bone adaptation in the femur

**DOI:** 10.1002/cnm.2633

**Published:** 2014-04-21

**Authors:** Diogo M Geraldes, Andrew T M Phillips

**Affiliations:** Structural Biomechanics, Department of Civil and Environmental Engineering, Skempton Building, Imperial College LondonLondon SW7 2AZ, UK

**Keywords:** femur, bone adaptation, orthotropic, isotropic, finite element modelling

## Abstract

Functional adaptation of the femur has been studied extensively by embedding remodelling algorithms in finite element models, with bone commonly assumed to have isotropic material properties for computational efficiency. However, isotropy is insufficient in predicting the directionality of bone's observed microstructure. A novel iterative orthotropic 3D adaptation algorithm is proposed and applied to a finite element model of the whole femur. Bone was modelled as an optimised strain-driven adaptive continuum with local orthotropic symmetry. Each element's material orientations were aligned with the local principal stress directions and their corresponding directional Young's moduli updated proportionally to the associated strain stimuli. The converged predicted density distributions for a coronal section of the whole femur were qualitatively and quantitatively compared with the results obtained by the commonly used isotropic approach to bone adaptation and with *ex vivo* imaging data. The orthotropic assumption was shown to improve the prediction of bone density distribution when compared with the more commonly used isotropic approach, whilst producing lower comparative mass, structurally optimised models. It was also shown that the orthotropic approach can provide additional directional information on the material properties distributions for the whole femur, an advantage over isotropic bone adaptation. Orthotropic bone models can help in improving research areas in biomechanics where local structure and mechanical properties are of key importance, such as fracture prediction and implant assessment. © 2014 The Authors. *International Journal for Numerical Methods in Biomedical Engineering* published by John Wiley & Sons, Ltd.

## 1. INTRODUCTION

Adaptation algorithms have been incorporated into finite element (FE) studies in many areas of biomechanics that focus on bone morphogenesis and response to altered loading conditions 1–7. Bone was initially assumed to be a self-optimising linearly elastic continuum that responded to changes in strain energy density (SED) 1,8,6,9. Coelho *et al.* 10 and Kowalczyk 11 have used SED as the driving stimulus for the optimisation of bone with a hierarchical macrostructural and microstructural description. However, SED can produce convergence problems during the adaptation process at a continuum level 4. The action of directional-dependent normal strains on the bone matrix has been put forward as the generator of physiological mechanobiological signals that activate osteocytes 13,12 and better suited as the driving stimuli of the adaptation process in continuum models 4,15,14.

In order to model the process of bone adaptation, the driving stimulus needs to be a physiologically meaningful representation of the *in vivo*mechanical environment 16. Therefore, the FE model of the bone being studied is required to be as close to the physiological state as is reasonably possible. This involves careful selection of its constitutive representation, mesh, geometry, loading and boundary conditions 17. FE models of the femur frequently fall short of these criteria. In order to simplify the analysis, 2D representations of the femur 15,19,18,11 and partial models 21,22,20,16,6,23,10,14 are commonly used and ignore the adaptation process in different planes or regions of importance. Artificial boundary conditions, such as restricting displacement at a distal end of the femoral shaft, induce stress concentrations around the restrained region 22,20,15,5,16,6,24,23,14. Non-physiological loading conditions such as applying hip contact forces (HCFs) and muscle forces as point loads 21,26,25,16,10,14 are often adopted for simplicity. These affect the strain and stress distributions in the surroundings of the load application point as well as in the trabecular bone beneath the cortex, so increased discretisation has been recommended 27. Phillips 28 proposed a free boundary condition approach to produce an equilibrated model of the femur, with the ligaments and muscles spanning across the hip and knee joints explicitly included. Balanced models such as this do not directly constrain the bone in a non-physiological manner 29,27 and produce reduced absolute strain values for the femur undergoing a single-leg stance 27,24, resulting in more physiological stress and strain distributions 30.

Bone is usually modelled with isotropic material properties in an attempt to reduce computational times 1,2,31, despite the anisotropic nature of the material properties being measured experimentally 32,34,33. Orthotropy has been shown to be the closest approximation to the bone's anisotropy, short of full anisotropic modelling 32. In addition, isotropy is insufficient in predicting the directionality of the observed microstructure of the bone 38,35–37.

The need for a physiological continuum model of the material properties distribution and structure orientation across the femur in order to understand its biomechanical behaviour has been emphasised 39. A review of the regression equations that have been fitted between elastic properties measured experimentally and computed tomography (CT) derived densities suggests that it is difficult to accurately determine this relationship 40. Furthermore, CT images are composed of scalar density values resulting from a combination of local porosity and tissue mineralisation and, therefore, are not able to predict the directionally dependent elastic properties of the bone required to model its structural directionality at a continuum level 41. Recent developments in micromechanics and X-ray physics 42 have allowed for extraction of orthotropic elastic properties from CT data. These studies rely on observer-dependent estimations of the trajectories of the principal material directions from the bone's geometry and from recognisable collagen structures amongst volumetric CT data of varying resolution 44,45,43.

Consequently, an iterative strain-driven orthotropic 3D bone adaptation algorithm was developed as part of the presented study. Orthotropic orientations of each element were aligned with the local principal stress directions, following *Wolff's Law* 38,46,37. Directional material properties were updated proportionally to the local strain stimuli, according to the *Mechanostat* theory for bone adaptation 47, which has been shown to have a logical biochemical basis 48–50.

The orthotropic adaptation algorithm proposed was compared against the commonly used isotropic approach. Both approaches were applied to a fully balanced loading configuration with the muscles and ligaments spread along their attachment sites. Boundary conditions that do not constrain the femur in non-physiological ways were adopted. The predicted density distributions for a coronal section of the whole femur were qualitatively and quantitatively compared with the results obtained by the commonly used isotropic approach to bone adaptation as well as *ex vivo* imaging data. The study investigates how important the contribution of orthotropic adaptation can be in the production of bone models where directionally dependent mechanical properties are required to be physiologically represented. We hypothesise the following: (1) the orthotropic approach will produce material distributions closer to that observed *in vivo*; and (2) additionally, orthotropy can produce reliable information about the local structural composition of the bone.

## 2. METHODS

### 2.1. Finite element model

The FE model of the femur was built in Abaqus/CAE (v. 6.12, SIMULIA, Dassault Systèmes SA, Vèlizy-Villacoubly, France). The external geometry of the femur was extracted from CT scans of a third-generation Sawbones synthetic femur (Pacific Research Laboratories, Inc., Vashon, WA, USA) and meshed with 326026 four-node linear tetrahedral elements. These had a mean element edge length of 2.37mm. Mesh density and properties were within values found to be adequate for numerical modelling of the femur by a previous study 51. Sensitivity studies were also performed by the authors to assess the dependence of the predicted results on mesh properties 52. The femur was positioned with the centre of the femoral head coinciding with the origin of the global reference system and the *x*, *y* and *z* axes defined according to International Society of Biomechanics recommendations 53. The femur's positioning and orientation were extracted from HIP98 54, where kinetic and kinematic data were measured *in vivo* in combination with hip joint contact forces from instrumented prostheses. The peak frame of the fourth trial of the level walking load cycle for the subject HSR was selected as the load case to apply on the FE model (HSRNW4). The data produced from this subject's trials have been considered reliable by other studies 55,56. In order to equilibrate the femur during the static analysis without influencing muscle recruitment, the inertial action of the contralateral leg and the missing torso was considered 55. The inter-segmental moments and forces between the pelvis and the femur were extracted from the inverse dynamics analysis of the HIP98 kinematic data by an open-source musculoskeletal model validated against the same public dataset 55,57. These were then applied at the centre of the hip joint structure and can be seen in Table 1.

**Table 1:** The inter-segmental forces (%BW) and moments (%BW.m) extracted from the inverse dynamic analysis performed by Modenese *et al.* 55 for the peak frame of the trial HSRNW4 and applied at the hip joint centre.

Trial	Peak frame	*F*_*x*_	*F*_*y*_	*F*_*z*_	*M*_*x*_	*M*_*y*_	*M*_*z*_
HSRNW4	27	10.4	− 91.3	− 3.0	− 10.2	6.0	− 0.7

The body weight (BW) of subject HSR is 860 N.

**Figure 1:**
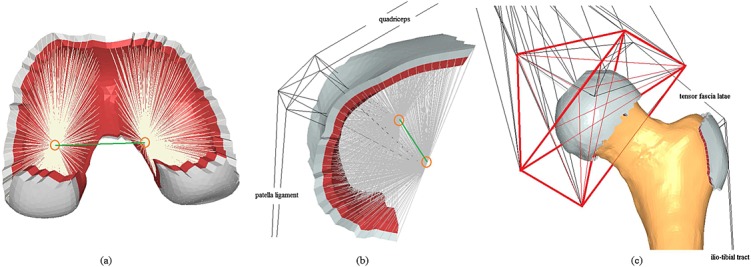
The artificial bi-layered structures (cortical bone, in grey, and cartilage-like elastic layer, in red) defined. The medial and lateral nodes along the joint's functional axis are highlighted in orange and the beam transferring the resulting moments between them in green at (a) the tibio-femoral joint and (b) the patello-femoral joint. The artificial truss structure defined at the hip joint (c) is highlighted in red.

Some modifications were made to the femur model to promote the transfer of loads across the hip, greater trochanter, tibio-femoral and patello-femoral joints back to the femur. Bi-layered structures composed of a 1-mm-thick internal isotropic elastic layer representing cartilage (*E* = 5MPa, *ν* = 0.49; Figure 1, in red) 58 and a 1-mm-thick external isotropic elastic cortical bone layer (*E* = 18GPa, *ν* = 0.3; Figure 1, in grey) 7 were projected from hand-picked contact surfaces for each of the three joints and modelled with linear six-node wedge elements. The external surface nodes of the bi-layered structures were connected to two nodes located medially and laterally along the functional axes for the tibio-femoral and patello-femoral joints (Figure 1, in orange) or at the centre of rotation for the hip via sets of linear beam elements with elastic properties similar to the cortical bone and a cross-sectional area of 10mm ^2^. The functional axes used for the tibio-femoral and patello-femoral joints were measured by Klein Horsmann in a cadaveric study 59 and were introduced in order to restrict the joints' movements to the flexion/extension plane, in a hinge-like fashion, a common assumption made in musculoskeletal models 55. At the condyles and the patella, the two nodes along the functional axis were connected via a beam element in order to transfer the moments between them (Figure 1, in green). The medial condylar node was fixed against displacement, allowing the femur to pivot about the tibial plateau at the tibio-femoral joint 60 (Figure 1(a)). Stiff two-node axial connector elements were included to create a hinge-like trapezoid structure, representing the region of the patella connected to the patellar ligament and the quadriceps and allowing for force transfer back to the femur (Figure 1(b)). At the hip joint, an artificial stiff truss structure connected the acetabular region to muscle insertion points on the pelvis, sacrum, lumbar spine and a point representative of L5S1, as proposed by Phillips 28 (Figure 1(c)). The hip was modelled as a pin joint with the inter-segmental forces and moments defined in Table 1 applied at its centre of rotation.

**Figure 2:**
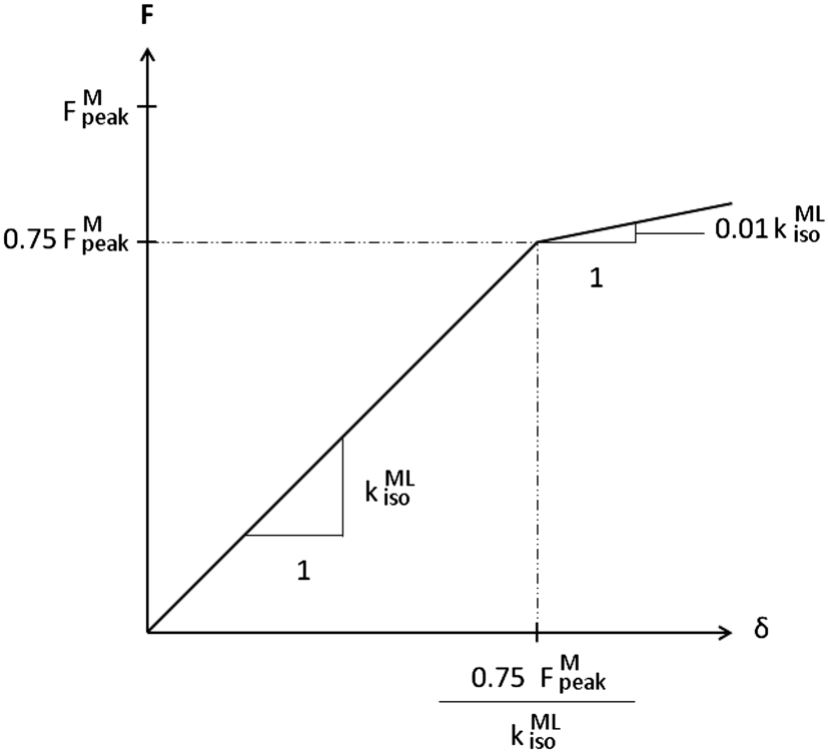
Force–displacement relationship implemented for each of the muscles, where 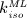 is the reference stiffness value and 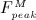 the peak contractile force.

**Figure 3:**
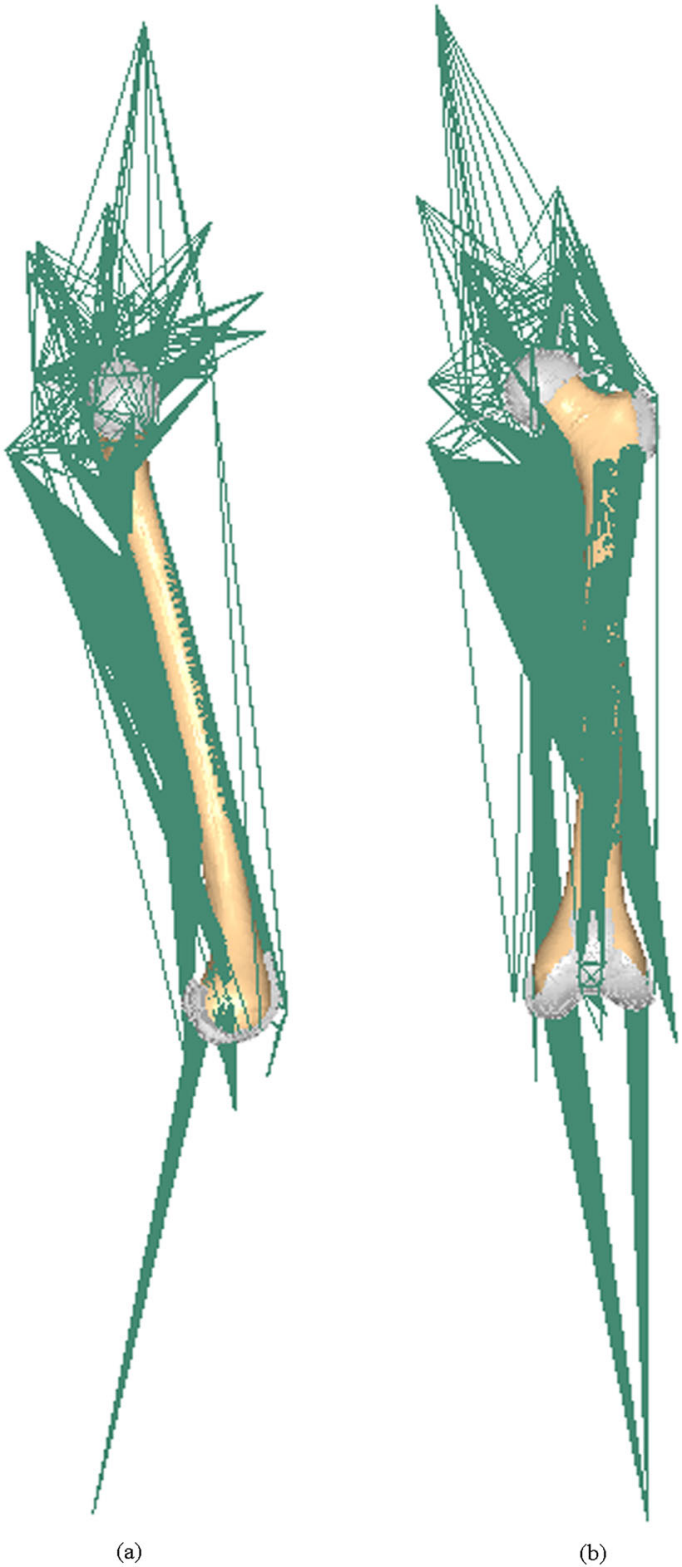
Medial (a) and anterior (b) views of the finite element model of the whole femur with muscles, ligaments and joints explicitly included.

A total of 26 muscles and seven ligamentous structures were explicitly included. Their origination areas as defined in the muscle standardised femur 61 were mapped onto the femoral mesh and the number of connectors, *C*, that formed each group was given by the number of nodes within these mapped areas. The insertion points in the pelvic and tibial regions were extracted from Phillips' 28 model of the femoral construct 28. The typical force–displacement curve for these connectors was defined by a reference stiffness value, 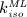, and a peak contractile force, 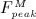, from 62 (Figure 2). The values for 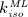 for each muscle group were calculated according to Equation 1, where 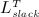 is the tendon slack length 63.

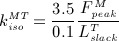
1 Stiffness was lowered after 0.75 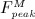, promoting activation of other muscle groups as the force generated by each muscle approaches its maximum 28. Given that muscles act in tension 63, stiffness values were considered to be negligible (0.01 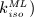 in compression. The properties of the muscles and ligaments included in the model are compiled in Table 2. Changes were applied to the definition of some muscles in order to represent their anatomical position and function more physiologically, in comparison with the model proposed by Phillips 28. An extra connector element spanning between the turning point at the greater trochanter and the insertion point at the tibia was included in the iliotibial tract structure. Its stiffness was also increased to 97N/mm, following tensile tests by Merican 64. The peak force for the patella tendon was increased to the 2500N measured as its ultimate failure load 65. The tensor fascia latae was modified to allow for wrapping around the greater trochanter and force transfer back to the femur. This region of the muscle contact was modelled as a bi-layered structure, similar to the joint structures described previously (Figure 1(c)). Fixed constraints were applied at the insertion points of the muscles and ligaments on the tibia and fibula 28. The point in space representing the insertion at the lumbar spine was connected to ground via a spring element with a stiffness of 10N/mm in the anterior–posterior direction and negligible stiffness in the other two directions (0.1N/mm), in order to simulate the balancing action provided by the upper torso during different gait activities 66. The resulting model with all the joints, muscles and ligamentous structures can be seen in Figure 3.

**Table 2:** Properties of the muscles and ligaments included in the model: peak contractile forces 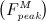, tendon slack length 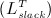 and reference stiffness values 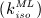.

Muscle	*F*_*peak*_^*M*^ (N) 62	*L*_*slack*_^*T*^ (mm) 63	*k*_*iso*_^*MT*^ (N/mm)	*C*
Adductor brevis	285	20	499	127
Adductor longus	430	110	137	300
Adductor magnus caudalis	220	150	51	63
Adductor magnus cranialis	880	150	205	1396
Biceps femoris long head	720	341	74	1
Biceps femoris short head	400	100	140	299
Gastrocnemius lateralis	490	385	45	55
Gastrocnemius medialis	1115	408	96	148
Gemeli	110	39	99	77
Gluteus maximus	1300	132	345	406
Gluteus medius	1365	61	783	120
Gluteus minimus	585	31	660	99
Gracilis	110	140	98	1
Iliopsoas	430	90	177	50
Pectineus	175	20	306	92
Piriformis	295	115	90	28
Psoas	370	130	100	1
Quadratus femoris	225	24	328	37
Rectus femoris	780	346	79	2
Sartorius	105	40	92	1
Semimembranosus	1030	359	100	1
Semitendinosus	330	262	44	1
Tensor fascia latae	155	425	13	1
Vastus intermedius	1235	136	318	2578
Vastus lateralis	1870	157	417	695
Vastus medialis	1295	126	360	330
Gluteal iliotibial tendon	720	N/A	85	1
Iliotibial tract	430	N/A	97[Table-fn tf2-1]	2
Patella tendon	2500[Table-fn tf2-2]	N/A	1000	2
Anterior cruciate	N/A	N/A	200	13
Lateral collateral	N/A	N/A	100	26
Medial collateral	N/A	N/A	100	28
Posterior cruciate	N/A	N/A	200	17

The stiffness values were distributed by the number of connectors defined for each group, *C*. N/A, not applicable.

a From Merican *et al.* 64.

b From Stäubli *et al.* 65.

### 2.2. Adaptation algorithms

The complete femur model presented earlier was loaded and submitted to one of two different optimisation processes: orthotropic or isotropic adaptation.

#### 2.2.1. Orthotropic adaptation

At each iteration, the stress, ***σ***_*ij*_, and strain, ***ε***_*ij*_, tensors were extracted for each element and post-processed in MATLAB (v. R2007b, MathWorks, Natick, MA, USA). An eigenanalysis of the stress tensor gives the local principal stress vectors, *σ*_*pv*_, and corresponding principal stress values, *σ*_*p*_ (Equation 2).

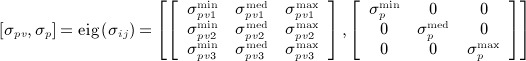
2 The element orthotropic material orientations were rotated to match with the calculated local principal stress orientations, following Wolff's trajectory theory 38,67 and previous work for 2D 15,68,52, in agreement with proven optimum orientations for orthotropic materials undergoing a single load case 69. The three local orthotropic axes, *x*_1_, *x*_2_ and *x*_3_, were associated with the minimum, 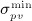; medium, 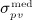; and maximum, 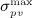, principal stress vectors, respectively. The local strain stimulus associated with the transformed orthotropic material axes, 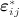, was calculated for each element according to Equation 3 68,52.

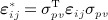
3 The material properties were adjusted in order to bring the local strains within the remodelling plateau, as proposed in the *Mechanostat* theory for bone adaptation 47 A normal target strain, *ε*_*nt*_, of 1250 μstrain with a margin of μ_0_ = ± 0.2*ε*_*nt*_, was used to define this plateau. Each iteration, the Young's modulus, 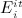, of the elements outside the remodelling plateau was updated proportionally to the absolute value of the associated normal local strain stimulus (Equation 4), and limited between 10MPa and 30GPa 41.

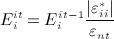
4 Shear moduli, 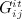, were taken to be a fraction of the mean orthotropic Young's moduli 15,68,52 (Equation 5).

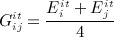
5 Poisson's ratios for each element, 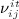, were assumed to be less than or equal to 0.3 70,71. In order to satisfy the thermodynamic restrictions on the elastic constants of the bone, some elements' Poisson's ratios were altered, ensuring that the compliance matrix remained always positive definite (Equation 6) 67.

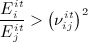
6 If 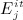 was greater than 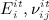 was kept at 0.3, while 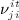
was adjusted such that the equality constraint was maintained (Equation 7).

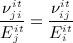
7 A ‘dead zone’ of elements was defined in order to exclude elements of low elastic stiffness and undergoing negligible strains from the applied convergence criteria. An element was considered to be in the ‘dead zone’ when the maximum absolute normal strain values, 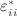 were less than 250 μstrain and, simultaneously, its directional Young's moduli, 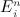, were all below 100MPa. The adaptation process was considered to achieve a state of convergence when the average change in Young's moduli of all elements outside the ‘dead zone’ was less than 2% between successive iterations. All elements were assigned the same initial orthotropic elastic constants (*E*_1_ = *E*_2_ = *E*_3_ = 3000 MPa,*ν*_12_ = *ν*_13_ = *ν*_23_ = 0.3,*G*_12_ = *G*_13_ = *G*_23_ = 1500 MPa) and local orthotropic orientations matching the global femoral axis system. A convergence study was performed to confirm that the model had a limited range of sensitivity to this arbitrary starting point 52.

#### 2.2.2. Isotropic adaptation

Because isotropic symmetry does not require any information on direction of the material properties, Young's modulus for each element, *E*^*it*^, was updated proportionally to the maximum absolute value of the principal strains according to Equation 8.

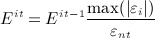
8 Similar to the orthotropic adaptation, the isotropic Young modulus values were updated to bring the local strains within the plateau of ± 0.2*ε*_*nt*_ around the same normal target strain, *ε*_*nt*_, and were limited between 10MPa and 30GPa. Based on the isotropic assumption, *G*^*it*^ was taken as Equation 9.

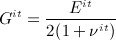
9 Poisson's ratio for each element, *ν*^*it*^, was assumed to be equal to 0.3. A ‘dead zone’ was also defined, and the same convergence criteria for the orthotropic adaptation was applied. All elements were assigned the same initial isotropic constants (*E* = 3000 MPa,*ν* = 0.3,*G* = 1500 MPa).

### 2.3. Imaging data

The results produced by both the orthotropic and isotropic adaptations were compared with *ex vivo* imaging data of two femur specimens.

A CT scan of an ethically obtained specimen of a male cadaveric femur, 55years old, weight 94kg and height 188cm, was taken with a SOMATOM Definition AS+ scanner (Siemens AG, Munich, Germany) based in the Queen Elizabeth Hospital, Birmingham, UK. The specimen was scanned at 120kV and 38.0mAs with an effective spatial resolution of 0.71mm. The density and normalised density greyscales of the coronal slice of the whole femur were compared with the predictions for isotropic and orthotropic adaptations.

In addition, micro-CT ( μCT) data for the proximal region of a male cadaveric femur, 27years old, weight 75kg and height 175cm, were also ethically obtained. The specimen was scanned using a HMXST 225 CT cone beam system with a 4MP PerkinElmer Detector (Nikon Metrology, Tring, UK) based in the Natural History Museum, London, UK, at 145kV and 150 μA and with an effective spatial resolution of 78.7 μm. The directionality of the trabecular structures of a coronal section of the femoral head was compared with the dominant orthotropic orientations.

## 3. RESULTS

Table 3 summarises the predicted values obtained for the resultant components (*F*_*r*_, *F*_*x*_, *F*_*y*_ and *F*_*z*_) of the HCFs for both converged models in %BW. The isotropic model produced higher resultant HCFs than the orthotropic model (332% against 319%). The isotropic model took three fewer iterations to converge than the orthotropic model (26 vs 29). Both models achieved the pre-determined convergence criteria, with an oscillating behaviour observed beyond 10 iterations as they attempted to reach the remodelling plateau.

**Table 3:** Predicted values obtained for the resultant components (*F*_*r*_, *F*_*x*_, *F*_*y*_ and *F*_*z*_) in (%BW) of the hip contact forces for the isotropic and orthotropic models.

Forces	Material	*F*_*x*_	*F*_*y*_	*F*_*z*_	*F*_*r*_
Predicted	Isotropic	59	− 319	73	332
	Orthotropic	53	− 306	71	319

For the converged solutions of the orthotropic and isotropic iterative simulations, the density of each element was calculated using a modulus–density relationship measured for the trabecular bone in the femoral neck 72 (Equation 10).

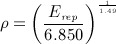
10
*E*_*rep*_ is the representative Young modulus for each element and is calculated according to Equation 11 for the orthotropic case. For the isotropic model, *E*_*rep*_ is taken as the isotropic Young modulus, *E*.

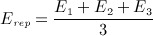
11 The maximum relative density value achieved in the iteration process was 2.41g/cm ^3^. The average representative Young's modulus and density for both models are shown in Table 4. The converged isotropic model was 60% stiffer and 25.4% denser than the orthotropic model.

**Table 4:** Average values for representative Young's modulus (*E*_*rep*_, in MPa), average density (*ρ*, in g/cm ^3^) and isotropic–orthotropic ratio for the converged isotropic and orthotropic models.

	Average *E*_*rep*_ (MPa)	Average *ρ *(g/cm^3^)
Isotropic	4306	0.572
Orthotropic	2689	0.456
Ratio	1.60:1	1.24:1

**Figure 4:**
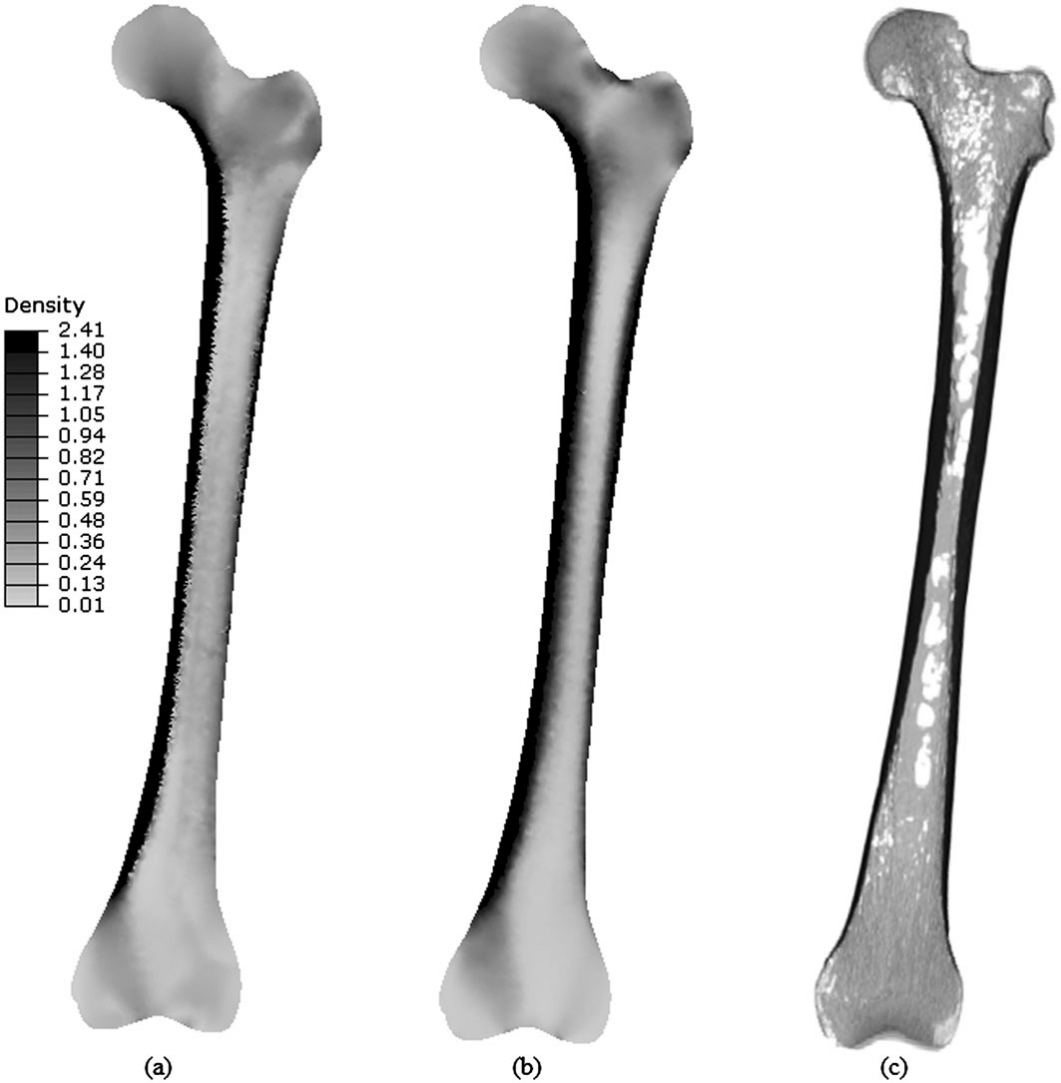
Coronal slices of the predicted density distributions (g/cm ^3^) for the (a) isotropic and (b) orthotropic models; (c) coronal slice of a CT scan of the whole femur.

Figure 4 shows the coronal slice representations of the density distributions resulting from the isotropic (a) and orthotropic (b) adaptation processes for the converged femur. All elements with density above 1.4 g/cm ^3^ were grouped together as dense cortical bone, in order to allow for better visualisation of the predicted density distributions for the trabecular bone. These were compared with a coronal slice taken from the CT scan (c) of the whole femur.

**Figure 5:**
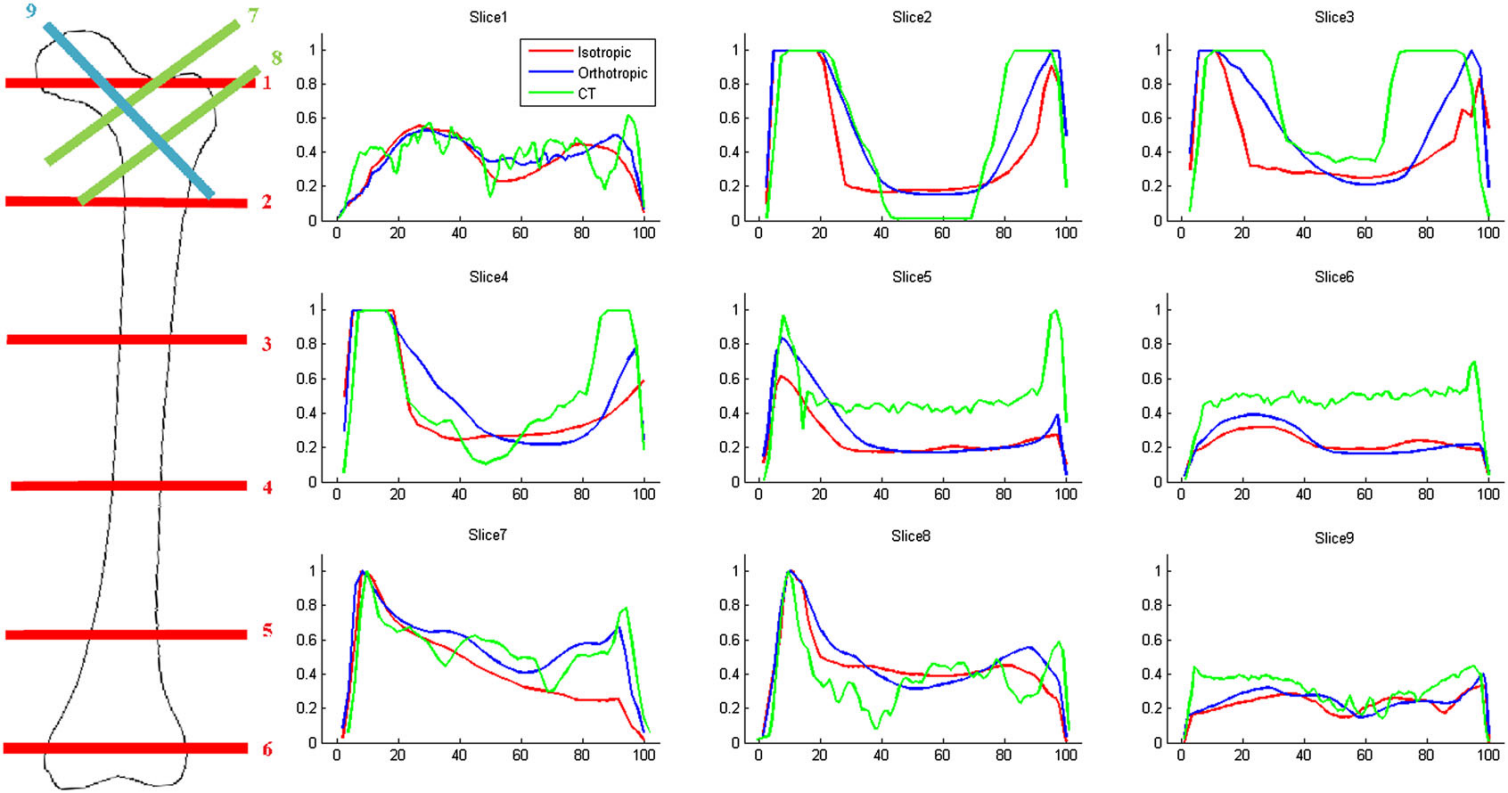
Greyscale profiles for nine slices across the femoral head (1, 7, 8 and 9), shaft (2, 3 and 4) and condyles (5 and 6) for the coronal representation of the density distribution for the converged isotropic (red) and orthotropic models (blue) and CT scan (green) of the whole femur. The density profiles are normalised between 0 and 1 along the percentage width of the slices taken.

Figure 5 shows the greyscale profiles of nine slices across the femoral head and neck region, shaft and condyles (transverse slices taken at 5%, 20%, 40%, 60%, 80% and 95% of the length of the femur) for the three coronal representations of the density distributions seen in Figure 4. These profiles were extracted using ImageJ 73, normalised and plotted between 0 and 1 (the maximum relative density value) along the percentage width of the slices taken.

The root mean squared error (RMSE, %) and Pearson's product-moment coefficient (*r*) between the two different predictions and the CT scan profiles can be seen in Table 5. These were calculated for the first third (medial aspect, 0–33%), the last third (lateral aspect, 66–100%) and the whole width (0–100%) of the slice. The average values for the three main regions and the whole femur are also processed.

**Table 5:** The root mean squared error (RMSE, %) and Pearson's product-moment coefficient (*r*, *p* < 0.0001) between the two different predictions (isotropic and orthotropic) and the CT scan profiles, for the first third (0–33%), the last third (66–100%) and the whole width (0–100%) of the slice.

Slice	Region	Model	0–100%		0–33%		66–100%	
			RMSE (%)	*r*	RMSE (%)	*r*	RMSE (%)	*r*
1	5% femoral head	Iso	32.48	0.49	17.31	0.77	22.90	− 0.12
		Ortho	29.23	0.49	17.08	0.77	20.74	− 0.02
2	20% shaft	Iso	75.83	0.74	43.09	0.77	57.81	0.59
		Ortho	51.27	0.88	25.92	0.88	38.92	0.72
3	40% shaft	Iso	107.50	0.29	65.73	0.37	78.23	− 0.66
		Ortho	82.32	0.54	35.04	0.72	64.87	− 0.09
4	60% shaft	Iso	63.95	0.67	28.38	0.86	55.40	0.37
		Ortho	64.03	0.65	36.38	0.89	48.41	0.74
5	80% shaft	Iso	72.34	0.53	34.80	0.73	53.69	0.60
		Ortho	68.29	0.46	27.07	0.69	51.83	0.81
6	95% shaft	Iso	66.15	0.43	30.57	0.85	42.81	0.64
		Ortho	66.10	0.25	21.43	0.89	45.24	0.80
7	Neck	Iso	25.65	0.72	18.53	0.89	17.21	0.68
		Ortho	12.29	0.88	9.56	0.93	5.38	0.89
8	Greater trochanter	Iso	26.67	0.58	22.48	0.82	12.47	− 0.14
		Ortho	30.72	0.55	26.08	0.81	14.90	− 0.13
9	Femoral head	Iso	30.06	0.40	23.60	0.46	16.81	0.26
		Ortho	25.87	0.50	19.31	0.40	15.98	0.24
	Femoral head	Iso	28.72	0.55	20.48	0.73	17.35	0.17
		Ortho	24.53	0.60	18.01	0.73	14.25	0.24
	Femoral shaft	Iso	82.43	0.57	45.73	0.67	63.81	0.10
		Ortho	65.87	0.69	32.45	0.83	50.73	0.46
	Femoral condyles	Iso	69.25	0.48	32.69	0.79	48.25	0.62
		Ortho	67.20	0.35	24.25	0.79	48.54	0.80
	Whole femur	Iso	55.63	0.54	31.61	0.72	39.70	0.25
		Ortho	47.79	0.58	24.21	0.77	34.03	0.44

The average values for the three main regions and the whole femur are also included.

The converged orthotropic model predictions were in general found to be closer to the CT greyscale profile than the converged isotropic model. The orthotropic predictions resulted in lower RMSE and higher Pearson's *r*, particularly for the lateral side of the slice widths, indicating a better correlation with *in vivo* measurements. Poorer predictions (*r* < 0.5) were produced in the femoral condyle region for both models. Both material symmetry assumptions produced good results for the medial cortical shaft, whilst the lowest correlations between profiles were found in slices 1, 6 and 9, cutting through regions largely composed of trabecular bone.

**Figure 6:**
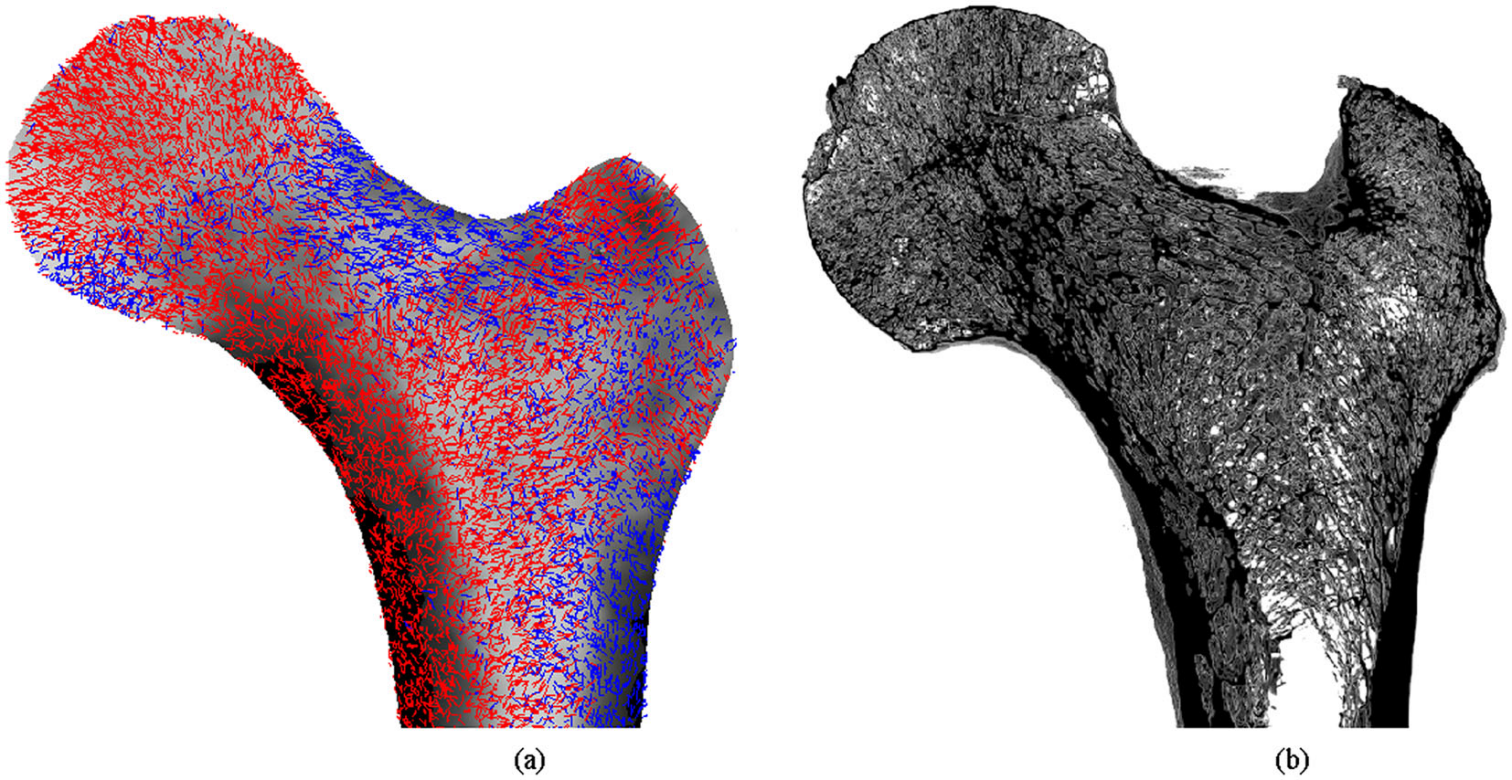
Dominant material orientations produced by the orthotropic bone adaptation superimposed with density distributions for coronal sections of the proximal femur (a) and compared with μCT reconstructions of the same regions (c). The dominant directional Young moduli are highlighted: *E*_1_ (red) and *E*_3_ (blue).

For the orthotropic model, the dominant material directions were defined as the orientation associated with the highest directional Young modulus for each element. These were projected onto the density distributions for coronal representations of the proximal femur (Figure 6(a)) and compared with a 5-mm-thick reconstruction of μCT slices of the same region (Figure 6(b)).

## 4. DISCUSSION

The loading environment of the FE model has been shown to be a key stepping stone in the attempt to produce a physiologically meaningful driving stimulus for the adaptation process 16,14. Table 3 shows that both isotropic and orthotropic approaches produced relatively small differences between HCFs, agreeing with other comparisons between mechanical environments for FE models with isotropic and orthotropic material properties 74,75. The HCFs predicted were higher than the values measured *in vivo* for the same instance of the gait cycle 54. However, computationally derived HCFs are often over-predicted in comparison with measurements from instrumented prosthesis 28,55.

Figure 4 shows a comparison between the isotropic (a) and orthotropic (b) model predictions and a coronal slice of the CT scan of the whole femur specimen (c) for the same region. These predictions show good agreement with the structures produced by similar 2D 1,15,19 and 3D adaptation studies 20,23 of the proximal femur. Many important density features observed in the CT scans were correctly predicted by both approaches, such as the low-density regions in the intra-medullary canal, Ward's and Babcock's triangles, the region just above the principal compressive group in the femoral head and medial to the superior part of the femoral neck and the epicondylar regions for both condyles. However, the isotropic assumption did not accurately represent the density distribution for a coronal slice, as it did not predict the dense cortical distribution along the lateral shaft, superior aspect of the femoral neck and the greater trochanter. The orthotropic predictions of the thick femoral shaft and the denser regions in the superior and inferior regions of the femoral neck, along the surface of the greater trochanter and around the intercondylar notch are seen to be coherent with the CT scan. The use of a modulus–density relationship (Equation 10) to produce the density plots in Figure 4 is limiting because it is a specimen-dependent empirical relationship obtained for the femoral neck. Because this relationship is used for both the isotropic and orthotropic model results, the comparison between them still holds. More rigorous approaches have been put forward 44,43 and would allow for a more accurate representation of the density distribution in the femur.

The orthotropic assumption produced more defined Ward's and Babcock's triangles. It also resulted in a less-stiff and lower-mass optimised femur, under the same loading conditions (Table 4). Predictions of greyscale profiles for nine slices across the femoral head, shaft and condyles show that the orthotropic predictions have higher Pearson's *r* coefficients for the lateral shaft and the slices across the femoral neck. The RMSE value is generally lower for the profiles predicted with orthotropy compared with the isotropic assumption. However, in regions where mainly trabecular bone is present (such as slices 6 and 9, across the condyles and femoral head, respectively), the profiles show poor agreement compared with the CT scans. These are regions where trabeculae have been proposed to adapt to the shear stresses arising from complex loading scenarios 76,37. The inclusion of a shear modulus adaptation algorithm could, therefore, have a positive impact in the density distribution predictions. It would also overcome the limitation of the *ad hoc* assumption of taking shear moduli to be a fraction of the mean orthotropic Young moduli.

A further advantage of using orthotropic material properties instead of isotropic symmetry is that directionality of the bone material properties can also be predicted. The proposed continuum approach we present in this study circumvents the assumption of using a pre-defined library of microstructure geometry 11 because it allows the system to optimise the combination of material orientations in order to provide the minimum energy solution for the load case it is subjected to. The adaptation algorithm proposed attempts to represent the underlying behaviour of both the cortical and trabecular bone structures as an orthotropic continuum with optimised material properties and orientations, whilst representing the complete femur with all muscle groups and ligaments explicitly modelled and load applicators included to promote physiological load application, with the possibility of being developed for multiple load cases in the future. Figure 6 shows the dominant orthotropic axes predicted by the algorithm (a) in comparison with a μCT reconstruction for a similar coronal slice of the proximal and distal femurs (b). The directionality of all main trabecular groups documented was predicted for the proximal femur 36: (i) the principal compressive group, composed of stiff, densely packed trabeculae arching from the medial cortex of the shaft towards the articular surface; (ii) the secondary compressive group arising from the medial cortex of the shaft, right below the principal compressive group, and curving towards the superior region of the femoral neck and the greater trochanter; (iii) the principal tensile group, composed by trabeculae arching from the lateral cortex across the neck of the femur and ending in the inferior part of the femoral head; (iv) the secondary tensile group, originating in the lateral cortex just inferiorly to the principal tensile group, arching towards the mid-line of the upper end of the femur; and (v) the greater trochanter group, made by poorly defined trabeculae along the greater trochanter region. These arise as a structural response to the necessity to transfer load along the femur from an oblique to vertical direction 77,35,76. Some other interesting features were also satisfactorily predicted. The cortical thickness seems to be a good match with the one observed in the μCT slice, and trabeculae can be seen radiating from the centre of the femoral head and meeting its superior surface at perpendicular angles 78,35.

Despite the encouraging results, there are several limitations to the current models and methods. The geometry of the FE model of the femur was representative of neither the geometry of the subjects from which HCFs were measured nor the specimens used to obtain the CT and μCT scans. Subject-specific hip geometry has been found to influence the calculation of the HCFs 79. The definition of certain muscles as straight paths of action and the inter-segmental forces used could have contributed to the overprediction of the HCFs 55. These reasons may explain some of the discrepancies between the calculated forces and the ones measured *in vivo* 54. It can also give a justification for some of the differences observed in the predicted density distributions and trabecular directionality. The displacement restrictions implemented at the medial condyle node introduced artificial constraints to movement in the distal part of the equilibrated FE model, possibly resulting in estimation errors of the loading environment and, therefore, influencing the predictions for this region. The modelling of surface contact at the hip and knee joints could induce a more physiological behaviour of the model.

It is estimated that the average person undertakes 1.1 million walking cycles a year 80. The load case selected corresponds to the peak of the level walking load cycle, where contact forces exceeding 250% body weight going through the femur have been measured *in vivo* 54. This load case has been used in several FE and remodelling studies with good results for the proximal region of the femur 2,3,21,26,16,14 but may accentuate the differences between the isotropic and orthotropic models. The use of a single load case is a significant limitation when describing the complex mechanical environment the femur is subjected to physiologically 81,37, particularly in the distal region, also adapted to higher flexion load cases 76. Reduced wall thickness in the anterior and posterior aspects of the cortical shaft may be a result of the simplified loading applied. Further work should include more load cases for a variety of frequent daily activities in order to improve the prediction of the distribution of the mechanical properties and associated orientations, particularly in the distal part of the femur and across the femoral head. These are regions that have evolved to resist the shear stresses arising from the multiple load cases the femur undergoes 78,35,76,37. Inclusion of more daily activities may result in an increase in the shear stresses in the femur, with the resistance of the trabecular structures to shear potentially playing a more important role than in the single load case model 37. Therefore, a shear modulus adaptation algorithm will be included in future studies. The proposed model results in an optimised structural system to most efficiently resist the loads generated by the instance of the walking cycle with highest contact forces, much like the trabecular structure that has been thoroughly studied in the proximal femur 35–37. Time and frequency dependence of the orthotropic adaptation process would need to be considered if the model were to be extended to the study of bone remodelling around implants and bone fractures.

The topological density distributions predicted by the model seem to be in reasonable agreement with data extracted from the CT scan. The directionality generated at the continuum level was comparable with μCT slices along the coronal plane for the proximal femur region. It also showed to be coherent with published studies of the trabecular features of these regions 38,78,35,36. We believe the method proposed in this study can provide an alternative way of assigning orthotropic material properties to continuum models of bone, with material orientations aligned to resist the principal stresses arising from the loading the femur is subjected to.

It is tentatively concluded that the orthotropic assumption is more advantageous in comparison with the isotropic material symmetry assumption, confirming the initial hypothesis of the study outlined. Orthotropy provides a more accurate representation of bone's elastic symmetry and can also give information about the three-dimensional directionality of bone's tissue-level material properties. The use of a balanced model allows for the prediction of the adaptation process for the whole femur, without artefacts induced by the application of fixed boundary conditions directly on the bone in question. An orthotropic model for the complete 3D femur has been produced. The inclusion of multiple load cases and of a shear modulus adaptation algorithm could further improve the predictions. A robust orthotropic continuum model of the whole femur has potential in achieving a more thorough understanding of bone's structural material properties, thus improving the knowledge we have of its mechanical behaviour and response to the various loading environments it may be subjected to. Such a model could contribute to the improvement of the design of orthopaedic implants and fracture fixation devices, providing information on the directional properties of the bone surrounding these devices and how it may adapt to the changing mechanical environment.
